# Surfactant-Based Chemical Washing to Remediate Oil-Contaminated Soil: The State of Knowledge

**DOI:** 10.3390/toxics12090648

**Published:** 2024-09-04

**Authors:** Yanxin Zhao, Yuhuan Sun, Haihan Sun, Fang Zuo, Shaoping Kuang, Shuwu Zhang, Fayuan Wang

**Affiliations:** College of Environment and Safety Engineering, Qingdao University of Science and Technology, Qingdao 266042, China

**Keywords:** soil washing, petroleum pollution, oil-contaminated soil, remediation technologies, surfactant

## Abstract

As the energy demand increases, there is a significant expansion and utilization of oil resources, resulting in the inevitable occurrence of environmental pollution. Oil has been identified as a prevalent soil contaminant, posing substantial risks to the soil ecosystems. The remediation of soil contaminated with oil is a formidable undertaking. Increasing evidence shows that chemical washing, a remediation technique employing chemical reagents like surfactants to augment the solubilization, desorption, and separation of petroleum hydrocarbons in soil, proves to be an efficacious approach, but the latest advances on this topic have not been systematically reviewed. Here, we present the state of knowledge about the surfactant-based chemical washing to remediate oil-contaminated soil. Using the latest data, the present article systematically summarizes the advancements on ex situ chemical washing of oil pollution and provides a concise summary of the underlying principles. The use of various surfactants in chemical washing and the factors influencing remediation efficiency are highlighted. Based on the current research status and knowledge gaps, future perspectives are proposed to facilitate chemical washing of oil-polluted soil. This review can help recognize the application of chemical washing in the remediation of oil-polluted soil.

## 1. Introduction

Oil pollution is the consequence of oil leakage, seepage, or emission into the earth’s surface or bodies of water, leading to detrimental effects on the environment. Petroleum exerts its influence on diverse ecosystems and biota, encompassing both aquatic and terrestrial environments. The release and discharge of petroleum entail the presence of toxic chemicals, thereby posing an imminent and direct hazard to both the environment and human well-being [1]. The ramifications of oil pollution are extensive, encompassing soil degradation, contamination of water sources, disruption of marine ecosystems, and the loss of wildlife [2,3]. Consequently, oil pollution engenders broad and far-reaching consequences, placing at risk the stability and sustainability of various ecosystems. The accumulation and magnification of oil in biota within marine and terrestrial environments can lead to long-term chronic toxicity to human health [4].

**Figure 1:**
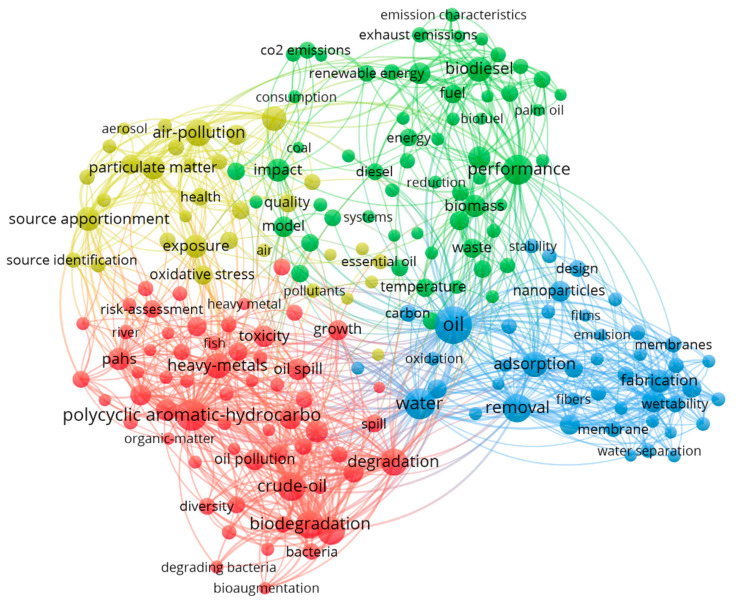
Visualization of research hotspots on oil pollution. Data source: Web of Science Core Collection; Search terms: “Oil pollution”; Search time frame: 1 January 2017 to 11 April 2023.

In this paper, we utilized VOS viewer software (Version 1.6.18, https://www.vosviewer.com/contact, accessed on 9 March 2023) to analyze and create a visualization map of the research literature on oil pollution. The purpose of this analysis was to systematically summarize and integrate the current state of research and hotspots in the field of oil pollution. Figure 1 displays the co-occurring keyword map on “oil pollution”, which includes 7734 retrieved documents. The figure shows four clusters. The red cluster pertains to oil pollution and soil, the yellow to the atmosphere, the blue to water, and the green to topics like performance, biodiesel, and biomass.

## 2. Soil Oil Pollution

**Figure 2:**
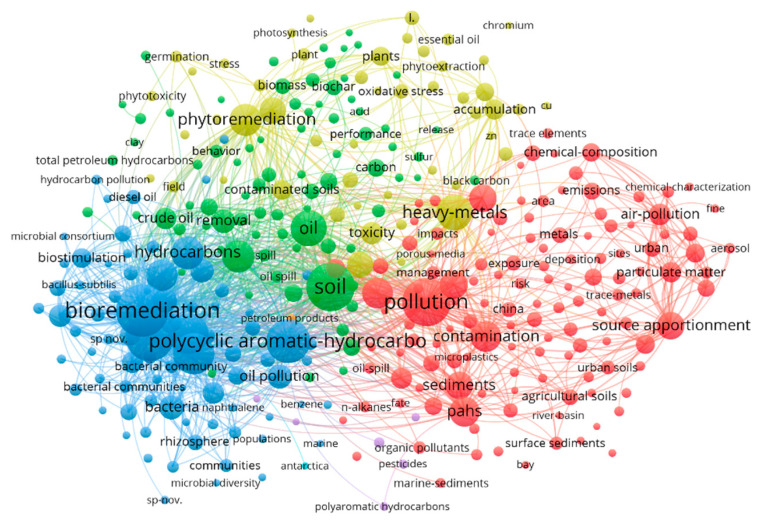
Visualization of research hotspots on soil oil pollution. Data source: Web of Science Core Collection; Search terms: “Oil pollution” and “Soil”. Search time frame: 1 January 2017 to 11 April 2023.

We added “soil” as a search term, leading to 1986 relevant articles, and generated Figure 2 to show the co-occurrence map of keywords with a frequency over 50 times, including four major clusters and three minor clusters. The green cluster is associated with remediation, the red cluster with water and atmosphere, the yellow cluster with topics such as plants and toxicity, and the blue cluster with biota. Notably, “bioremediation”, “polycyclic aromatic hydrocarbons”, “heavy metals”, and other keywords are common in the study of petroleum oil pollution in soil, but they do not include “soil”, “oil”, or “pollution”.

### 2.1. The Sources of Oil in Soil

Soil petroleum hydrocarbons have two primary sources. The first source comes from petroleum leakage and emissions during production, transportation, processing, and use. The second source results from human production and activities, including combustion emissions like automobile exhaust, fertilizer and pesticide use, and industrial waste gas. Leakage and emission of petroleum create pollution sources with higher concentrations of petroleum hydrocarbons than combustion emissions [5].

### 2.2. The Harm of Oil to Soil Ecosystems

Petroleum hydrocarbons are among the primary pollutants causing soil contamination. They significantly alter the physical and chemical properties of soil such as its texture, structural state, minerals, and heavy metal concentrations, which together adversely impact biological activity [2,6]. Modification of one or more of the soil’s chemical characteristics may have a direct or indirect unfavorable effect on its chemical fertility. Petroleum hydrocarbon pollutants have a detrimental impact on soil enzyme activity, limiting organic mineralization [7]. The hydrocarbon organic groups in such pollutants have the potential to react with nitrogen and phosphorus present in soil by forming organic nitrogen and organic phosphorus, thereby hindering nitrification and phosphorus removal from the soil resulting in a reduction in available nitrogen and phosphorus levels, ultimately leading to a loss of soil fertility. Furthermore, the organic matter in petroleum hydrocarbons dramatically increases the organic carbon content in the soil, effectively altering the carbon-to-nitrogen ratio and affecting soil fertility [8]. Additionally, crude oil spills further worsen environmental conditions by increasing toxic metal levels in the surrounding area [9].

Petroleum has a diverse array of impacts on plant life, including hindering their uptake of water and mineral salts, disrupting plant metabolism, causing a lack of chlorophyll and nutrients, making plants more susceptible to pests and diseases, and resulting in poor growth and root deformation [10,11]. Hydrocarbon compounds not only affect the plants directly, but also exhibit genotoxicity [12]. Furthermore, crude oil does not only affect plant growth, but also induces oxidative stress in earthworms, inhibiting the activities of crucial enzymes such as superoxide dismutase and catalase [7]. Crude oil exposure drastically affects soil microorganisms’ respiration and phospholipid content, and high concentrations of crude oil can significantly lower the carbon dioxide and phospholipid phosphate (PLP) content in soil samples (*p* < 0.05) [3]. Finally, when the amount of petroleum hydrocarbons reaches 0.5%, it has a detrimental effect on luminescent bacteria activity [13].

## 3. Remediation of Oil-Contaminated Soil

**Figure 3:**
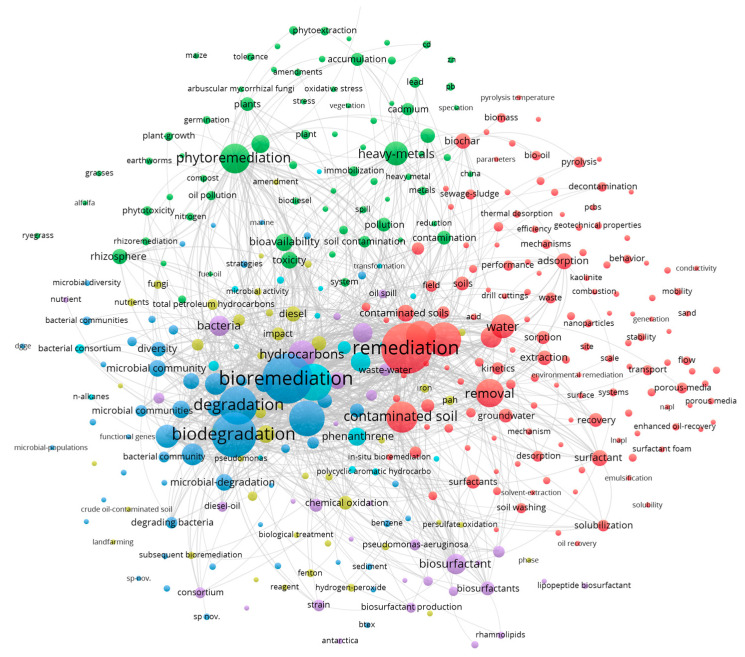
Visualization of research hotspots on oil-contaminated soil remediation. Data source: Web of Science Core Collection; Search terms: “Oil” and “Soil” and “Remediation”. Search time frame: 1 January 2017 to 11 April 2023.

There are many mature technologies that already exist, such as bioremediation, chemical oxidation, electrokinetic remediation, solvent extraction, thermal remediation, and combined remediation. The required conditions and effects of each remediation technology are different. Table 1 lists various remediation technologies for oil-contaminated soil. A total of 2330 documents were gathered by searching for “oil, soil, remediation” in the subject, abstract, and keywords. A keyword co-occurrence map of the retrieved results is shown in Figure 3, displaying the most common subjects of investigation for petroleum-contaminated soil. The blue cluster in the figure reveals that the most widely researched topic is bioremediation. This is followed by remediation technologies (shown in red), which consist of keywords such as “soil washing, surfactant, extraction, adsorption”, etc. The green cluster is related to plant-based remediation, and the purple cluster indicates the prominence of “biosurfactant”. Other remediation systems and pollutant types are represented in the yellow and light blue clusters, respectively. From the keyword co-occurrence map, it is evident that the most commonly used and popular techniques for oil pollution remediation presently include bioremediation (microbial and phytoremediation), soil washing, chemical oxidation, and thermal desorption.

## 4. Soil Washing

As shown in Figure 2 and Figure 3, bioremediation is the most extensively studied soil remediation technique. However, in practical applications, ex situ soil washing is widely used to remove pollutants from highly-contaminated soils [27,28]. This preference is due to the ability of soil washing techniques to achieve effective results at a lower cost and within a shorter time frame.

Chemical cleaning of petroleum hydrocarbon in soil is a process that uses chemical agents to enhance the removal of hydrocarbons from contaminated soil. There are different types of chemical agents that can be used for cleaning petroleum hydrocarbon in soil, such as surfactants, oxidants, solvents, and chelating agents.

There exist several types of soil-washing solvents used to eliminate petroleum hydrocarbons from contaminated soil, which include organic solvents, surfactants, supercritical and subcritical fluids, and other novel materials [29]. Sui et al. [30] illustrated that petroleum ether extracted 76–94% of total petroleum hydrocarbons (TPHs) from soil in only 20 min, indicating that it can be used as an effective extractant. 

Research has demonstrated that by utilizing supercritical carbon dioxide extraction, the residual oil content of soil can be reduced to 0.2 wt% [31]. In contrast, liquefied gas extraction utilizes liquefied hydrocarbons like butane and tetrafluoroethylene as extraction media at low temperatures and pressures, with the capacity to extract oil content from the soil, hence serving as an alternative to supercritical carbon dioxide extraction technology [32].

In one study, Kim et al. [28] employed core cross-linked amphiphilic polymer (CCAP) nanoparticles to purify petroleum-contaminated silty soils while comparing the effectiveness of CCAP nanoparticle cleaning with two nonionic surfactants (Triton X-100 and Brij 30). The tests indicated that CCAP exhibited superior cleaning efficiency with a maximum of 96.2% (compared to Brij 30: 74.8%; Triton X-100: 51.4%), and this purification method led to a clearer aqueous solution compared to nonionic surfactants.

**Figure 4:**
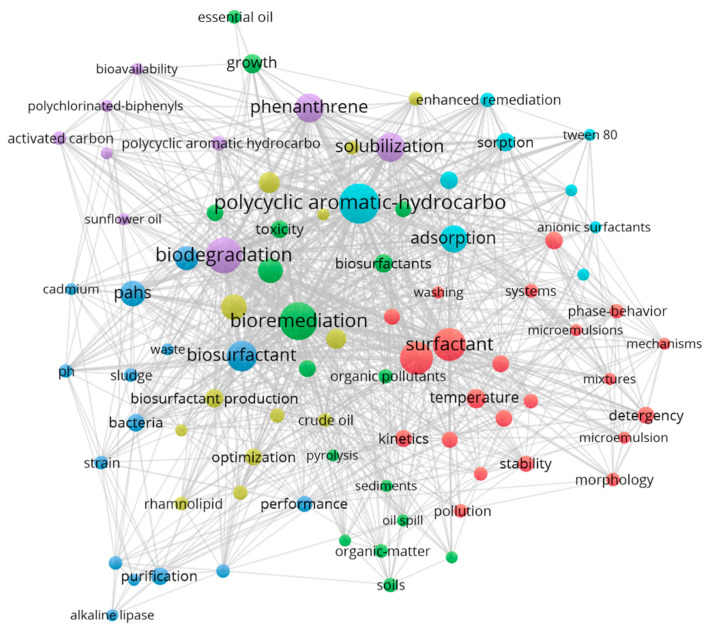
Visualization of research hotspots on soil washing techniques for oil pollution. Data source: Web of Science Core Collection; Search terms: “Oil” and “Soil” and “Washing”. Search time frame: 1 January 2017 to 11 April 2023.

As shown in Figure 4 presenting the research hotspots regarding petroleum-contaminated soil washing technologies from a perspective view, the frequently recurring key topics on cleaning and remediation have been identified, including but not limited to: “Bioremediation”, “Biodegradation”, “Surfactants”, and “Biosurfactants”. It is worth noting that soil-washing technologies are often utilized in combination with bioremediation techniques. Here, soil washing is employed as an initial treatment stage to quickly reduce pollution concentrations on-site and simultaneously provide a favorable soil environment for the subsequent bioremediation treatment. Thus, both remediation methods are maximized for effectiveness. For example, Fanaei et al. [26] achieved optimal results by using biosurfactant-assisted soil washing combined with H₂O₂ to stimulate the biodegradation of heavy oil. Additionally, the use of surfactants is also widely recognized in soil-washing processes.

As summarized in Table 1, various remediation technologies have been developed to remediate oil-contaminated soil. However, these techniques each have their own disadvantages, such as being time-consuming, high-cost, causing secondary pollution, and being difficult to operate. For example, petroleum ether and other organic solvents have toxic effects on the soil environment and can lead to secondary pollution. Additionally, supercritical extraction technology is limited in its application due to high cost and safety issues stemming from the required high-pressure support. While newly developed cleaning materials may offer higher remediation effectiveness than traditional surfactants, most of the material research remains in an immature state, and the potential risks associated with their actual use are unclear. In comparison, surfactants are frequently implemented in soil washing due to their low cost, ease of operation, and safety, without necessitating high-pressure or high-temperature equipment.

### 4.1. Surfactant

Surfactants are substances that can reduce the surface and interfacial tension of liquids, making them useful for solubilizing, emulsifying, dispersing, and wetting. Typically, surfactants consist of a hydrophilic group (such as a carboxyl or sulfate group) and a hydrophobic group (such as a long-chain hydrocarbon), giving them amphiphilic properties. As a result of their amphiphilic properties, surfactants can form a monolayer or micelle structure at the water–oil interface, resulting in a change in the interfacial state. Based on the charge type of the hydrophilic group, surfactants can be classified into four types: anionic, cationic, nonionic, and amphoteric. Table 2 lists the different types of surfactants used to wash petroleum hydrocarbons from soil.

#### 4.1.1. Mechanism of Surfactant Cleaning of Petroleum Hydrocarbons in Soil

**Figure 5:**
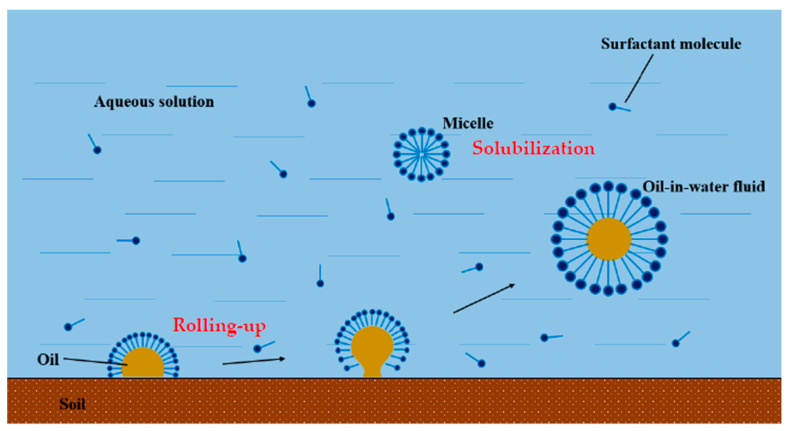
Diagram of surfactant cleaning of oil in soil.

Surface active agents have been shown to effectively increase the solubility and mobility of petroleum hydrocarbons in water, thus facilitating their removal from soil. Consequently, the use of surfactants for cleaning up petroleum hydrocarbons in soil is an effective remediation approach. Two mechanisms of surfactant-enhanced soil washing are the rolling-up and solubilization mechanisms, which are illustrated in Figure 5. The rolling-up mechanism refers to the process by which surfactants reduce interfacial tension, thereby facilitating the separation of contaminants from the surface of soil particles. In contrast, the solubilization mechanism involves surfactants forming micelles in solution, encapsulating contaminants and thereby increasing their concentration in the aqueous phase.

At surfactant concentrations lower than the critical micelle concentration (CMC), the surfactant monomers adsorb between soil particles and petroleum hydrocarbons, increasing the contact angle between them. The increase in contact angle causes soil particles to become more hydrophilic and less attractive to petroleum hydrocarbon molecules, which promotes the separation of pollutants from soil particles. This is known as the rolling-up mechanism.

At surfactant concentrations greater than or equal to the CMC, micelles with hydrophilic head groups and hydrophobic tail groups form in the aqueous phase. These micelles can solubilize and enclose petroleum hydrocarbon molecules, increasing their solubility and mobility in the water phase and promoting their migration from soil. This is known as the solubilization mechanism.

#### 4.1.2. Classification and Selection of Surfactants

##### Cationic Surfactants

Cationic surfactants primarily work through their cationic charge, such as quaternary ammonium compounds. They are characterized by high water solubility and strong stability. For instance, Abo-Riya et al. [39] synthesized two novel cationic copolymer surfactants while designing quaternary ammonium ion polymers. AISE4 had a CMC of 0.0001 mol/L and a surface tension of 28.5 mN/m, while AISM4 had a CMC of 0.0002 mol/L and a surface tension of 29.5 mN/m, all determined by measuring in seawater. Furthermore, cationic polymer surfactants exhibit high conductibility, stable foam height, good emulsifying properties, and are efficient in petroleum performance. Nevertheless, most soil surfaces bear negative charges, resulting in adsorption onto cationic surfactants with positive charges, which reduces the surfactant’s cleaning efficiency. As a result, the application of cationic surfactants in soil cleaning is limited.

##### Anionic Surfactants

A variety of anionic surfactants have been created due to their low cost, high interfacial activity, superior heat resistance, and desirable properties such as low toxicity, biodegradability, low critical micelle concentration, and electrolyte tolerance. For instance, Liu et al. [40] synthesized alkyl ethoxy sulfonate sodium (AEOSHS) using alcohol ethoxylates to recover heavy oil. AEOSHS is capable of producing a W/O emulsion with a high elastic modulus and low additional resistance. Thus, it can traverse through porous media at a lower displacement pressure, increasing the sweep efficiency of the oil displacement system. Compared with traditional surfactants such as SDS, AEOSHS can raise recovery rates by 18.17% and enhance efficiency by 9.46% [40].

##### Nonionic Surfactants

Anionic surfactants only change the hydrophilicity of oil droplets by adsorbing onto their surface. Conversely, nonionic surfactants can adsorb onto the surface and interior of oil droplets, disturbing the accumulation structure of resins or asphaltenes [41]. Various types of nonionic surfactants, including alkyl polyglucoside (APG), fatty acid glycerides, polyols (sorbitan esters, fatty alcohol esters), polyethylene oxide (long-chain fatty acid esters, fatty alcohol esters), and polyethylene oxide-polypropylene oxide copolymers, are commonly used. Zhang et al. [41] demonstrated that nonionic surfactants are instrumental in promoting crude oil viscosity reduction and demulsification via molecular dynamics simulation. Among them, the nonionic surfactant nonylphenol ethoxylate (NP-4) decreases the viscosity of heavy crude oil by increasing the surface hydrophilicity of oil droplets and disturbing the interior accumulation structure of droplets. Studies have shown that nonionic surfactants have destabilizing effects on intractable asphaltenes. Additionally, Han examined the use of the nonionic green surfactant APG for washing weathered crude oil-polluted soil and achieved optimal cleaning efficiency of 97% [35]. Furthermore, APG, derived from sustainable resources such as fatty alcohols and sugars, is less toxic and biodegrades efficiently, preventing secondary pollution in practical applications [42].

##### Amphoteric Surfactants

Amphoteric surfactants consist of both positively and negatively charged groups, including phospholipids, amino acids, and betaines in their molecular structure. In alkaline solutions, they exhibit anionic surfactant properties, while in acidic solutions, they show cationic surfactant properties. Hong et al. [43] synthesized a new amphoteric surfactant, 2-hydroxy-3-(N,N-dimethyl-N-dodecylammonium) propoxy sulfated cellulose (GDCS), using the etherification reaction of sulfuric acid cellulose and glycidyltrimethylammonium chloride. Despite the existence of numerous amphoteric surfactants, limited products have been utilized in practical applications due to factors such as cost, performance, raw material availability, and reaction complexity.

##### Gemini Surfactants

Numerous novel surfactants with attributes such as high biodegradability, chemical stability, environmental safety, efficient solubilization, and ease of recovery have been developed to promote environmental protection. Gemini surfactants, with two or more hydrophilic and hydrophobic head groups, have become increasingly popular with researchers as ultra-active second-generation surfactants for homogeneous catalytic reactions, emulsification, solubilization, and other applications. Si et al. [44] synthesized a new boron-containing anion-nonionic surfactant (SYW), using 1,3-propanediol polyether (PPG), boric acid, maleic anhydride (MA), and sodium metabisulfite as raw materials. The esterification-sulfonation reactions resulted in a significant reduction in the interfacial tension. SYG was created by mixing SYW, oleic acid, and ethanolamine in a 3:1:1 ratio where the interfacial tension dropped from 25.6 mN/m to 0.07 mN/m. SYG had more negative charges than SYW, and its absolute value was 40 mV at the oil–water interface compared to SYW’s 20 mV at a concentration of 2500 mg/L. Therefore, SYG provided both theoretical and experimental backing for heavy oil extraction and transportation.

Tehrani-Bagha et al. [45] synthesized a new Gemini surfactant in which an ester bond acted as the spacer between two quaternary ammonium groups as the hydrophilic head and dodecyl acted as the hydrophobic tail. Although this surfactant had a toxic impact on aquatic organisms, its toxicity was lower than that of its hydrolysis products. It could not be easily extracted, thereby decreasing the overall toxicity. Abo-Riya and Baker [46] used ethylenediamine, alkyl halides, and 3-chloro-2-hydroxypropyl sulfonate sodium to synthesize a Gemini anionic sulfonate surfactant that displayed exceptional surface activity and petroleum dispersibility in water solutions of varying salinity. It was thus well-suited for tackling petroleum pollution in oilfields.

##### Biosurfactants

Soil washing technology is hindered primarily by the solvent utilized, which can lead to secondary pollution. Certain cleaning agents have the potential to remain in the soil, leading to soil environment contamination due to their low biodegradability. The use of surfactants presents similar issues as they can be adsorbed into soil particles, ultimately affecting the efficiency of oil removal. Moreover, soil washing technology requires a large amount of solvents, resulting in high operating costs. The primary limitations of soil washing technology lie in selecting effective and environment-friendly solvents, and recovering/regenerating them. Biosurfactants can be a viable solution to traditional synthetic surfactants, due to their non-toxic, biodegradable, and ecologically-friendly nature. They are surface-active products produced through microbial metabolic processes, with examples such as glycolipids, polysaccharides, and lipopeptides. Also, biosurfactants are capable of maintaining stability in adverse and extreme environmental conditions, such as low/high temperatures, acidic/basic soils, and high salinity [47].

Urum et al. [48] investigated the effectiveness of synthetic and biosurfactants in removing crude oil. The biosurfactants used in the experiment were rhamnolipids and saponins, while the synthetic surfactant was sodium dodecyl sulfate (SDS). Soil contaminated with weathered oil was washed using a 20 mL surfactant solution at 20 °C for 20 min. The results showed both SDS and rhamnolipids had a similar efficiency of around 45%, followed by saponins at 27%. The results indicated that rhamnolipids and SDS are more effective in removing aliphatic hydrocarbons compared to aromatic hydrocarbons. In contrast, saponins are more effective in removing aromatic hydrocarbons. However, it is critical to note that producing biosurfactants is more costly compared to traditional synthetic surfactants.

### 4.2. Influencing Factors of Soil Washing

#### 4.2.1. Detergent Configuration

The remediation efficiency is influenced by both the soil environment and the cleaning agent’s properties. Therefore, it is crucial to comprehend the cleaning technology factors affecting remediation and to analyze their mechanisms for practical applications. Typically, a higher concentration of the surfactant translates to a more robust cleaning effect. However, the high concentration may lead to increased costs. Further, non-degradable residues resulting from the excess concentration may harm the soil. As the surfactant concentration increases, it results in molecular aggregation, thus producing micelles. The concentration of the surfactant required to form micelles is referred to as the CMC. The CMC value is dependent on several factors, including the type, structure, and composition of the surfactant, temperature, solution ion strength, and organic additives [49].

##### The Combination of Surfactants

Currently, a single combination of surfactants is no longer able to achieve the desired results. Continuous research has led to the development of new surfactant materials and various methods to enhance their efficiency. Mixing two or more kinds of surfactants can result in mixed micelles which exhibit synergistic effects, enabling them to possess higher solubilizing power and lower critical micelle concentration. This allows for a reduction in the usage of a single surfactant, leading to increased efficiency, reduced costs, and less environmental impact [50]. Compared to synthetic surfactants, the surfactant mixing process does not require complex synthetic conditions. For example, introducing nonionic surfactants into an anionic surfactant solution can reduce the precipitation of anionic surfactants with multivalent electrolytes, such as Ca^2+^ and Mg^2+^, thus reducing soil adsorption losses of anionic surfactants. In addition, the presence of anionic surfactants can also inhibit the adsorption of nonionic surfactants, thereby forming more micelles and increasing the efficiency of pollutant removal.

Gang et al. [51] demonstrated the synergistic effect of lipid-peptide biosurfactants and synthetic surfactants (petroleum sulfonate salts) in creating water-in-oil emulsions with good stability in a shorter period. One possible explanation can be ascribed to their synergism in reducing the interfacial tension between oil and surfactant solution. Han et al. [52], through dissipative particle dynamics (DPD) simulation, confirmed that the synergistic effect of anionic and cationic surfactant mixtures significantly reduced interfacial tension, causing the oil–water interface to change from laminar to emulsified state at lower surfactant concentrations. Furthermore, the weakening of interactions between anions and cations is affected by the steric hindrance of spacer groups. Zhou et al. [53] used a mixture of the anionic-nonionic surfactant alcohol ether sulfate (AES) and the cationic surfactant dodecyl trimethyl ammonium chloride (DTAC). The sulfate group in the AES molecule attracted DTAC molecules in the mixed surfactants, thereby reducing repulsion forces between molecules, and DTAC molecules enhanced the overall salt tolerance of the mixed surfactants.

##### Auxiliary Agents

To improve surfactant efficiency in cleaning petroleum hydrocarbons from soil, it is common to combine it with inorganic salts, alkalis, and other chemicals. Inorganic salts increase the concentration of electrolytes in the solution, reducing the critical micelle concentration required for surfactant micelle formation in water. This leads to an increase in micelle concentration and ability to encapsulate petroleum hydrocarbon molecules. On the other hand, alkalis help regulate the pH of the solution, bringing it closer to or above the pKa value of hydrocarbon molecules in the soil, thereby enhancing their solubility and fluidity [54].

For instance, Wei et al. [55] studied the impact of salinity on the micellar behavior of surfactants and the solubilization and desorption of pyrene. The results indicated that adding salt to surfactant solutions could decrease the interfacial tension, CMC, and surfactant adsorption on soil, while enhancing pyrene desorption in soil. In another study, Kumar and Mandal [56] investigated the effects of different types of surfactants, alkalis, and salts on changes in interfacial tension, emulsification, and wettability of crude oil. They found that the interfacial tension (IFT) value between crude oil and aqueous solution declined first and then rose with an increase in ion concentration in surfactants. Among them, the IFT value of cetyltrimethylammonium bromide (CTAB) decreased the most significantly. Adding sodium salt to nonionic surfactants reduced their IFT values because of weakened hydrogen bonding [57]. For ionic surfactants, the reduction in IFT was mainly due to the decrease in electrostatic repulsion between ion head groups. Alkalis could also react with acidic components in crude oil, producing surfactant inorganic salts or organic acid salts that are adsorbed at the oil–water interface, thereby further reducing interfacial tension.

Additionally, several other additives can enhance surfactant efficiency in oil removal. Wei et al. [58] demonstrated that the combination of biochar, rhamnolipids, and coated urea (N) had a synergistic effect and exhibited much higher efficiency than their individual application. Biochar increased the adsorption of aromatic compounds, whereas rhamnolipids and nitrogen enhanced the degradation of both heavy and light hydrocarbons. Chen et al. [59] developed a CO_2_-responsive microemulsion for treating petroleum-contaminated soil, achieving a significant reduction in oil content from 15 wt% to 1wt% due to its ultralow interfacial tension and high solubilizing ability.

#### 4.2.2. Factors Influencing the Chemical Washing Process

During the process of soil remediation, the efficiency of the remediation process is considerably influenced by environmental factors. While washing contaminated soil with surfactants, the final removal efficiency of pollutants can be influenced by various factors, including liquid-to-solid ratio, solution pH, stirring, temperature, and duration of the remediation period. 

##### pH Value

Fluctuations in pH levels can impact the solubility and dispersibility of surfactants, which ultimately affects the efficacy of cleaning. Typically, lower pH levels facilitate surfactant adsorption and dispersal, leading to enhanced cleaning effectiveness, but exceedingly low levels of pH may result in environmental damage. Incorporating sodium salts can fortify the cleaning efficiency of surfactants; however, it can also modify the pH level. The experiments by Huang et al. [38] showed that, within the range of pH levels 7 to 10, the removal rate of diesel amplified with an enhancement in pH for the surfactant SDS, but when the pH level reached 11, the removal rate substantially declined. In contrast, SDBS had the highest diesel removal speed at a pH level of 7. The pH levels needed for the maximum cleaning efficiency of various surfactants vary. The zeta potential of surfactants can also be influenced by pH levels. The SDS solution’s zeta potential attains its apex point at a pH level of 11, whereas that of SDBS occurs at pH levels 8 and 9. Post the inclusion of adjuvants, pH levels must be fine-tuned to accomplish optimal cleaning performance.

##### Temperature

**Figure 6:**
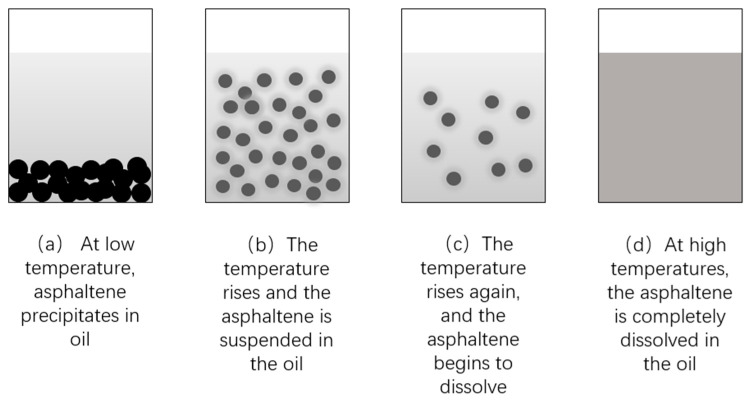
The schematic diagram depicts the precipitation, suspension, and dissolution of asphaltene in oil.

The performance of surfactants is significantly influenced by temperature. An elevated temperature increases the ability of surfactants to disperse and wet hydrocarbons, thereby accelerating the removal of petroleum hydrocarbons. Temperature not only has an impact on critical micelle concentration, surface tension, and emulsifying activity of surfactants [60], but also affects the solubility of asphaltene [61]. With an increase in temperature, asphaltene dissolves gradually in oil, reducing the viscosity of the oil fraction and improving the fluidity of oil, thereby affecting its interaction with surfactants, as shown in Figure 6. Based on the research of Li et al. [62], at a pressure of 30 MPa, when the temperature increased from 25 °C to 75 °C, the contact angle between the CO_2_-oil system increased from 77 °C to 90 °C which enhances the extracting and diffusion capabilities of CO_2_ at elevated temperatures. Nevertheless, exceedingly high temperatures significantly impact foam stability.

##### Mixing Mode

During ex situ cleaning, stirring is employed to enhance cleaning efficiency, with mechanical stirring being the most common method. This approach increases the contact surface area between the cleaning agent and contaminants, thereby improving the solubility and migration potential of petroleum hydrocarbons. However, mechnical stirring consumes energy and increases costs. Previous studies have shown that micro-nanobubbles can improve the removal of petroleum hydrocarbons [62,63,64,65]. Micro-nanobubbles, in addition to increasing surface area, reduce the surface tension of the cleaning solution, enhancing diesel’s dispersion and emulsification. Moreover, micro-nanobubbles create cavitation effects that break down petroleum adsorption layers on soil particles’ surfaces, ultimately promoting petroleum detachment and dissolution. Huang et al. [66] examined the enhanced effect of surfactant-based microbubbles during the cleaning of soil contaminated with diesel and found a 94.3% removal rate in the presence of microbubbles, which was 20.8% higher than that of conventional cleaning methods. Furthermore, the diesel solubility and migration rate improved by 0.16 g/L and 0.15 g/L, respectively, when microbubbles were present. Pang et al. [63] found that incorporation of O_3_ nanobubbles significantly increased the removal efficiency of diesel pollutants by surfactants from soil. The integration of micro-nanobubbles with surfactants provides an effective approach to remediate oil-contaminated soils. 

##### Other Conditions

Several studies indicate that the addition of sodium salts improves oil removal efficiency of surfactants. Nonetheless, high soil salinity reduces surfactant solubility and cleaning effectiveness. Additionally, high salinity can cause soil particle aggregation, thereby decreasing the solubility of petroleum hydrocarbons and negatively affecting cleaning efficiency. Soil washing time is time-dependent; an increased washing time improves removal efficiency until it reaches equilibrium, after which it may decrease [67]. Prolonging the washing requires extensive cost, necessitating optimization studies. The liquid-to-solid ratio is another crucial parameter in soil washing; higher ratios facilitate interaction between surfactants and pollutants, effectively trapping the latter. The removal efficiency is non-linearly proportional to the liquid-to-solid ratio. Despite its effectiveness, the larger equipment, higher costs, and generation of excess wastewater requiring further treatment must be considered when increasing the liquid-to-solid ratio [68].

#### 4.2.3. Factors Related to Soil Properties

Soil characteristics including particle size distribution, mineral composition, organic matter content, pH value, cation exchange capacity (CEC), and the presence of inorganic contaminants affect surfactant efficiency. Particularly, soil organic matter content and mineral composition have a significant impact on soil adsorption of surfactants.

As the organic matter content increases, soil adsorption for surfactants also increases, reducing the number of surfactants that form micelles [69]. Different types of clay minerals have diverse layered structures and charge distributions, which results in variations in their capacity to adsorb petroleum hydrocarbons and the efficiency of surfactant desorption [33]. As the clay mineral content rises, the adsorbed amount of surfactants also increases (from 5% to 20%) [70]. The removal of pollutants from soil is also affected by its pore size, shape, and porosity [71]. Soil components below 125 μm, as shown by Zhang et al. [72], contain more clay and organic matter that adsorb and precipitate surfactants, leading to the reduction in elution efficiency to approximately 40%. Nonetheless, separating soil particles first and then washing with a surfactant mixture can remove up to 80% of TPHs. Since soils and sediments usually carry negative charges that affect the adsorption of surfactants, charged surfactants with permanent and pH-dependent charges have varied adsorption properties depending significantly on the soil type. Anionic surfactants are adsorbed less due to their negative charge [73], while cationic surfactants have the highest adsorption due to electrostatic attraction between the surfactant and surface with similar alkyl chains. Anionic surfactants have reduced adsorption due to electrostatic repulsion [74]. Additionally, acidic pH conditions cause the positive charges carried by soil colloids to surpass the negative charges, resulting in higher adsorption of anionic surfactants by soil [75]. However, surfactant adsorption can limit the practicality of using surfactants to enhance the remediation effect, as it decreases the permeability of the water-containing layer. The effectiveness of soil properties for the removal of diesel from the soil, as identified by Li et al. [76], is based on sand content, anion exchange capacity, organic matter content, silt and clay content, specific surface area, and pH. Although not comprehensive, this ranking provides valuable reference values for the assessment of soil properties. For soils with high clay content, surfactant solutions generally have low penetration capacity. Due to the smaller particle size and larger specific surface area, contaminants are bound tightly to soil particles, making it challenging for surfactants to separate contaminants from the soil particle surfaces [77]. Generally, surfactant-based washing is not applicable to soils with clay content greater than 30%. 

#### 4.2.4. Parameters of Oil

The degree of difficulty in removing petroleum hydrocarbons from soil is influenced by several factors, including type, concentration, physicochemical properties, and degree of weathering. Different types of petroleum hydrocarbons have varying effects on surfactants; for instance, aromatic hydrocarbons have lower solubility and weaker impacts on surfactants, whereas alkanes have high solubility and more significant impacts on surfactants. However, polycyclic aromatic hydrocarbons (PAHs) pose a considerable challenge to removal, and appropriate surfactants must be selected accordingly for effective cleaning. Examples of surfactants that are effective in removing PAHs include the following.

Cationic surfactants are effective in cleaning PAHs owing to the relative polarity of the hydrocarbons. Cationic surfactants carrying a positive charge are exceptionally effective due to their polarity. For example, CTAB and hexadecyl trimethyl ammonium bromide (HTMAB) are two cationic surfactants that exhibit high cleaning efficiency for PAH-contaminated soil [78].

Polymeric nonionic surfactants have multiple hydrophilic and hydrophobic groups that can form abundant micelle structures, thereby increasing solubility and cleaning efficiency. Triton X-114 and TX-100 are examples of these surfactants that have demonstrated the capacity to remove PAHs. Adding to this, Jousse et al. [79] confirmed that Tween80 is an effective surfactant, demonstrating that toluene, an aromatic hydrocarbon, is more efficiently removed than n-decane, an alkane. 

The effectiveness of surfactants may be reduced by higher concentrations of petroleum hydrocarbons because surfactant molecules are limited in number and cannot efficiently wet and clean large quantities of hydrocarbons. Mixed hydrocarbons produce a synergistic effect that changes the cleaning effectiveness of surfactants. Additionally, the extent to which pollutants have aged impacts the efficiency of surfactant washing of soil. Pollutants that have undergone various physical, biological, and chemical processes in the soil and have strong soil-binding properties are more challenging to extract [80]. 

## 5. Conclusions and Future Perspectives

Soil pollution has a substantial impact on both human health and ecosystems. The article mentions several soil remediation technologies, whereas soil washing technology has been commonly utilized because of its effectiveness in remediating polluted soil. Surfactants, due to their ability to increase the removal of pollutants in soil, have become a prominent cleaning agent in soil washing technology. Consequently, with years of continuous research and exploration, surfactant-based washing technology in soil remediation has matured considerably.

Biosurfactants exhibit low toxicity, high biodegradability, and notable pollutant-removing effects. However, their production costs outweigh those of chemical surfactants. Recently, the gradual improvement of the efficiency of using surfactants in removing oil has been observed due to diverse new surfactants, combinations of various surfactants, micro-nanobubbles, and adding various adjuvants. Factors determining the effectiveness of soil remediation include surfactant properties, cleaning conditions, soil characteristics, and pollutants. It is necessary to develop and tailor efficient surfactants to be used in specific regions. To lessen the cost of surfactant-based soil remediation and further minimize soil pollution, both surfactant degradability and their recovery and reuse should be considered. Considering pollutant characteristics, site conditions, and practical operating conditions, combining several remediation methods is advised to fully utilize the advantages of various methods in terms of removal efficiency, remediation cost, and potential risks after remediation.

In summary, the development of surfactants is expected to concentrate on discovering more environmentally friendly, economical, and efficient surfactant cleaning strategies. The future priority should focus on new biodegradable surfactants that lessen the harm to soil and water ecosystems and enhance the cleaning procedure. Another area of concern is the implementation of surfactant cleaning techniques based on nanotechnology, which can lessen the quantity of surfactants and time needed to remediate petroleum hydrocarbon-contaminated soil. The development of efficient technologies to evaluate the efficiency and impact of surfactant cleaning technology will guarantee its safety and sustainability. The forthcoming surfactant cleaning strategies will prioritize ecological restoration and sustainability, have high specificity and effectiveness, and address the crucial mission of maintaining healthy ecosystems.

## Figures and Tables

**Table 1:** Remediation of oil-contaminated soil.

Remediation Technique	Technical Features	Process Details	Contaminant Conc.	Soil Characteristics	Process Duration	Maximum Efficiency Reported	Reference
Bioremediation	Environmentally friendly, low cost; highly time-consuming, low efficiency, greatly affected by environmental factors.	Composting stage (75 days) + vermiremediation stage (60 days); contaminated soil, lombricompost, rice hulls, and wheat stubbles (60:20:15:5% *w*/*w*); earthworm species *Eisenia fetida* and *Amynthas morrisi*	Diesel/3425 ± 50 mg/kg	Clay 20.5%; silt 59.5%; sand 20.0%; OM 1.5 ± 0.1%; ashes 0.8 ± 0.1%; TN 0.09 ± 0.01%; C/N 10; pH 6.0 ± 0.02	75 d + 60 d	60.81%	[14]
		*Pinus densiflora*, *Thuja orientalis*, and *Populus tomentiglandulosa* amended with microbial consortium and commercial compound fertilizer (NPK 21-17-17)	Diesel/6000 mg/kg	pH 5.65; EC 0.03 dS/m; OM 0.8%; CEC 1.9 cmol/kg	150 d	86.80%	[15]
Chemical oxidation	Highly efficient, low-cost, and easy to operate; potential risks and secondary pollution.	Oxidant: PMS; satalyst: nZVI; five serial applications of the 0.3% PMS/0.2% nZVI system	Diesel/6625 ± 115 mg/kg	Clay 8%; silt 10%; sand 82%; pH 4.2 ± 0.03; textural classes: loam; OM 4.51%; CEC 12.0 cmol/kg; EC 130.1 μS/cm; water content (*w*/*w*) 4.05%; TN 510 mg/kg	10 h	96.00%	[16]
		Oxidant: H_2_O_2_, persulfate; catalyst: Fe^2+^ (FeSO_4_); mechanical stirring with continuous addition of H_2_O_2_ of various concentrations using a peristaltic pump at ambient temperatures	Diesel/5000 mg/kg	Clay 12%; silt 47%; sand 41%; pH 5.7; textural classes: loam; OM 7.5%	40 h	80%	[17]
		Oxidant: H_2_O_2_; catalyst: zero-valent iron; mechanical stirring at 180 rpm in shaking water bath at 22 °C	Diesel/5030 ± 120 mg/kg	Clay 4.9%; silt 75.1%; sand 20.0%; pH 6.3; minerals: quartz, feldspar, kaolinite, goethite	3 h	90%	[18]
Electrokinetic remediation	High efficiency, low power consumption, strong controllability; not environmentally friendly, time-consuming.	Electric field: 1.0 V/cm, 2.0 V/cm; graphite electrode chambers 4 L using 0.03 mol/L citric acid	Commercial diesel fuel/11,680 mg/kg	Clay 47.24%; silt 42.44%; sand 3.17%; gravel 7.15%; TOC 1.70%; moisture 46.59%; EC 12.40 mS/cm; pH 7.8; carbonate < 0.1 mg/kg	15 d	73%	[19]
Solvent extraction	High efficiency, less time-consuming, wide applicability; a large amount of solvent consumption, potential hazards, and secondary pollution.	Anionic lipopeptide (LT) and nonionic sophorolipid (SL); concentration 100 mg/L; temperature 55 °C; ratio of sludge/liquid 1:3; stirring speed 300 rpm	Crude oil/17.79 wt%	Oily sludge; oil 17.79 wt%; water 3.54 wt%; solids 78.67 wt%	3 h	85%	[20]
		Rhamnolipid and sophorolipid; concentration 500 mg/L; temperature 45 °C; ratio of sludge/liquid 4:1; stirring speed 300 r/min; washing four times	Crude oil/45.66%	Oily sludge; water 42.37%; oil 45.66%	3 h	95.66%	[21]
Thermal desorption	High efficiency, fast, reliable; high cost, producing greenhouse gases, affected by high moisture content.	Microwave frequency heating; heating time of 30 min in a modified domestic microwave oven (power: 600 W; frequency 2.45 GHz; temperature: up to 275 °C)	Diesel fuel/1900 mg/kg	Fine sand; moisture content 10%; OM 3.55%; porosity 32.5%; pH 8.72; soil mineral: silica sand	1 h	90%	[22]
Combined remediation	Integrating the advantages of a variety of single techniques, the ideal remediation effect can be achieved; complex technological processes.	Current: 569 ± 2 mA/cm^3^; bottle-type dual-chamber MFC reactors with carbon fiber brush as anode and titanium wire mesh at 22 ± 2 °C for 140 days	Crude oil/24,085 mg/kg	Clay loam; pH 7.27 ± 0.08; EC 0.61 ± 0.01 mS/cm; sand 69.4%; silt 20.0%; clay 10.6%; TOC 28.5 ± 2.56 g/kg; TPHs 11.34 ± 3.26 g/kg; nitrate 1.90 ± 0.07 mg/kg; phosphate 1.91 ± 0.03 mg/kg; sulfate < 40 mg/kg	140 d	76.00%	[23]
		Predominant species of bacteria: *Pontibacillus*, *Sediminimonas*, *Georgenia;* Power: 132 ± 17 mW/m^2^; A cylindrical soil MFC with carbon cloth anode and activated carbon cathode for 182 days	Petroleum hydrocarbon/83,060 mg/kg	pH 8.26 ± 0.04; EC 5.45 ± 0.07 mS/cm; TN 93.11 ± 2.39 mg/kg; NH_4_^+^-N 1.60 ± 0.13 mg/kg; NO_3_^−^-N 1.19 ± 0.06 mg/kg; alkanes 48,751 ± 591 mg/kg; aromatics 27,947 ± 278 mg/kg; DON 21.60 ± 0.45 mg/kg; DOC 469.35 ± 0.15 mg/kg	182 d	52%	[24]
		Pyrolysis temperature of 400 °C and residence time of 30 min; N_2_ flow: 1 L/min continuous high purity (>99.999%); Additive: Fe_2_O_3_, Al_2_O_3_, K_2_CO_3_, CaO, HZSM-5, and red mud	Petroleum hydrocarbon/119 ± 5 g/kg	Sand 94.8%; silt 4.6%; clay 0.6%	30 min	>91.6%	[25]
		Mixed biosurfactant (surfactin + rhamnolipid) of 0.6 g/L, soil/water ratio of 20% *w*/*v*, temperature of 30 °C, and washing time of 24 h; the effluent was efficiently biotreated in the bioprocess with 5 g/L acclimate biomass daily stimulated with 0.1 mM H_2_O_2_	Petroleum hydrocarbon/32 g/kg	Clay loam; clay 32%; silt 38%; sand 30%; permeability 1.5 cm/h; moisture 4.63%; pH 7.2; TN 0.11%; TP 242.5 ppm; organic content 1.11%; density 1.96 g/cm^3^	18 d	99%	[26]

Abbreviations: CEC: cation exchange capacity; DOC: dissolved organic carbon; DON: dissolved organic nitrogen; EC: electrical conductivity; nZVI: nanoscale zero-valent iron; OM: organic matter; PMS: peroxymonosulfate; TN: total nitrogen; TOC: total organic carbon; TP: total phosphorus; TPH(s): total petroleum hydrocarbon(s).

**Table 2:** Remediation of petroleum hydrocarbons-contaminated soils by various surfactant solutions.

Type	Surfactant Conc.	Operating Condition	Contaminant Conc.	Soil Characteristics	Maximum Efficiency Reported	Reference
Nonionic	Triton X-100/150 mg/L	Mechanical stirring 160 rpm; liquid/solid 10:1; washing time 30 min; temperature 60 °C	Crude oil/20,000 mg/L	Clay soil; clay 13–24%; quartz 13–15%	68.00%	[33]
	Tween 80/4000 mg/L	Flow rate 0.01 mL/s; total amount of leachate 2000 mL	o-dichlorobenzene and p-dichlorobenzene/537.36 mg/kg	pH 7.87; TOC 20.17 g/kg; CEC 17.52 cmol/kg; specific gravity 1.98; sand 80.38%; silt 14.70%; clay 4.92%	68.00%	[34]
	0.75 wt% APG1214, 0.1 wt% Na_5_P_3_O_10_, 0.06 wt% Na_2_CO_3_, and 0.04 wt% Na_2_SiO_3_	Mechanical stirring 350 rpm; temperature 80 °C; washing time 30 min; solution/soil ratio 10 mL/g	Crude oil/123 mg/g	Clay 6%; silt 16%; sand 78%; pH 8.1; TOC 6.33%	97.00%	[35]
	Triton X-100/2.5% *v*/*v*; NaM-si/2.5% *w*/*v*; MWCNT/0.04% *w*/*w*	Mechanical stirring 220 rpm; washing time 7 days	Engine oil	Clay 18%; silt 75%; sand 7%	91.83%	[36]
	Tween20/30 mg/L	Mechanical stirring 160 rpm; liquid/solid 10:1; washing time 30 min; temperature 60 °C	Crude oil/20,000 mg/L	Clay soil; clay 13–24%; quartz 13–15%	91.30%	[33]
	Polyoxyethylene sorbitol hexaoleate/12 mg/L in phosphate buffer 960 mg/L	Mechanical stirring 275 rpm 48 h	PAH (C10–C24)/95 mg/kg	Sand 83%; silt 14%; clay 3%	50.00%	[37]
	APG/4000 mg/L	Flow rate 0.01 mL/s; total amount of leachate 2000 mL	o-dichlorobenzene and p-dichlorobenzene/537.36 mg/kg	pH 7.87; TOC 20.17 g/kg; CEC 17.52 cmol/kg; specific gravity 1.98; sand 80.38%; silt 14.70%; clay 4.92%	69.00%	[34]
Anionic	SDS/2.5% *v*/*v*; NaM-si/2.5% *w*/*v*; MWCNT/0.04% *w*/*w*	Mechanical stirring 220 rpm; washing time 7 days	Engine oil	clay 18%; silt 75%; sand 7%	92.22%	[36]
	Dodecyl methylnaphthalene sulfonates/400 mg/L	Mechanical stirring 160 rpm; liquid/solid 10:1; washing time 30 min; temperature 60 °C	Crude oil/20,000 mg/L	Clay soil; clay 13–24%; quartz 13–15%	86.30%	[33]
Cationic	CTAB/300 mg/L	Mechanical stirring 160 rpm; liquid/solid ratio 10:1; washing time 30 min; temperature 60 °C	Crude oil/20,000 mg/L	Clay soil; clay 13–24%; quartz 13–15%	<50%	[33]
Biosurfactant	Saponin/0.2 g/L	Water/soil ratio 10:1; temperature 45 °C; magnetic stirrer 340 rev/min; washing time 15 min	Diesel oil	pH 7.28; CEC 93.7 mol/kg; organic carbon 2.48%; sand 15.74%; clay 4.51%; silt 76.28%	61.70%	[38]
	Saponin/4 g/L	Flow rate 0.01 mL/s; total amount of leachate 2000 mL	o-dichlorobenzene and p-dichlorobenzene/537.36 mg/kg	pH 7.87; TOC 20.17 g/kg; CEC 17.52 cmol/kg; specific gravity 1.98; sand 80.38%; silt 14.70%; clay 4.92%	p-dichlorobenzene 76.34%; p-dichlorobenzene 80.43%	[34]
	Saponin/4000 mg/L	Flow rate 0.01 mL/s; total amount of leachate 2000 mL	o-dichlorobenzene and p-dichlorobenzene/537.36 mg/kg	pH 7.87; TOC 20.17 g/kg; CEC 17.52 cmol/kg; specific gravity 1.98; sand 80.38%; silt 14.70%; clay 4.92%	80.00%	[34]
	Anionic lipopeptide (LT) and nonionic sophorolipid (SL)/100 mg/L	Temperature 55 °C; ratio of sludge/liquid 1:3; stirring speed 300 rpm; washing time 3 h	Crude oil/17.79 wt%	Oily sludge; oil 17.79 wt%; water 3.54 wt%; solids 78.67 wt%	85%	[20]
	Rhamnolipid and sophorolipid/500 mg/L	Temperature 45 °C; ratio of sludge/liquid 4:1; stirring speed 300 r/min; washing four times; washing time 3 h	Crude oil/45.66%	Oily sludge; water 42.37%; oil 45.66%	95.66%	[21]

## Data Availability

No new data were created or analyzed in this study. Data sharing is not applicable to this article.
